# Supplementary material for: PSGL-1 inhibits HIV-1 infection by restricting actin dynamics and sequestering HIV envelope proteins
Source: Cell Discov. 2020 Aug 4;6:53. doi: 10.1038/s41421-020-0184-9 (PMC7400672; doi:10.1038/s41421-020-0184-9)
Supplement: Supplementary file 1 — Supplementary information [file 41421_2020_184_MOESM1_ESM.pdf]

Supplementary information

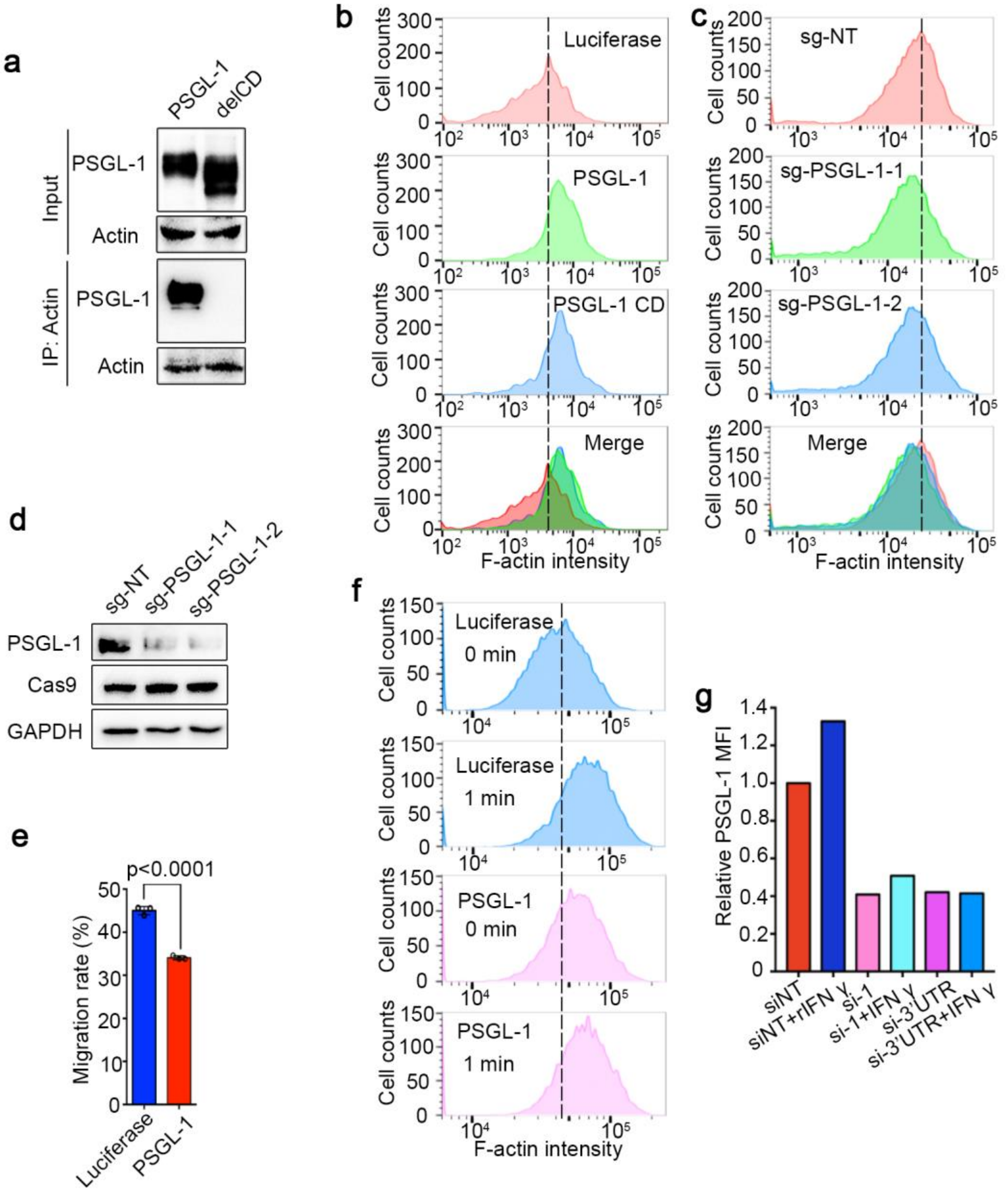

Supplementary information, Fig. S1 PSGL-1 stabilizes cellular F-actin. a MAGI cells transfected with plasmids expressing PSGL-1 or PSGL-1 delCD for 48 h before the cells were immunoprecipitated with anti-actin antibody and protein A agarose beads. Samples were then analyzed using Western botting. **b** Jurkat cells overexpressing luciferase, full length PSGL-1 or PSGL-1 cytoplasmic domain (CD) were fixed and stained with phalloidin. The intensity of phalloidin was quantitated with FACS. **c**, **d** PSGL-1 knockout Jurkat cells lines were either validated

by Western blotting (**d**) or stained with phalloidin for F-actin intensity measurement by FACS (**c**). **e** Jurkat cells overexpressing luciferase or PSGL-1 were stimulated by SDF-1 for 2 h in a transwell device. **f** Jurkat cells overexpressing luciferase or PSGL-1 were stimulated with SDF-1 or mock treated for 1 min and immediately fixed and stained with phalloidin for F-actin intensity measurement by FACS. Migrated cells were collected and counted by FACS. **g** FACS quantification of PSGL-1 expression level of IFN-γ treated and electroporated primary CD4+ T cells in **Fig. 1d and 1e**.

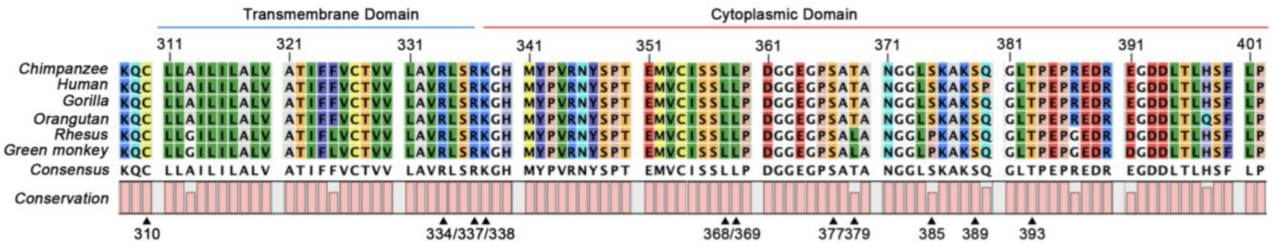

Supplementary information, Fig. S2 Sequence alignment of PSGL-1. Sequence alignment of the Transmembrane domain and cytoplasmic domain (CD) of PSGL-1 from six primate species. Triangles indicate the residues that were mutated to examine the sequence requirement for actin binding or gp41 binding or mutations that abolished dimerization of gag colocalization.

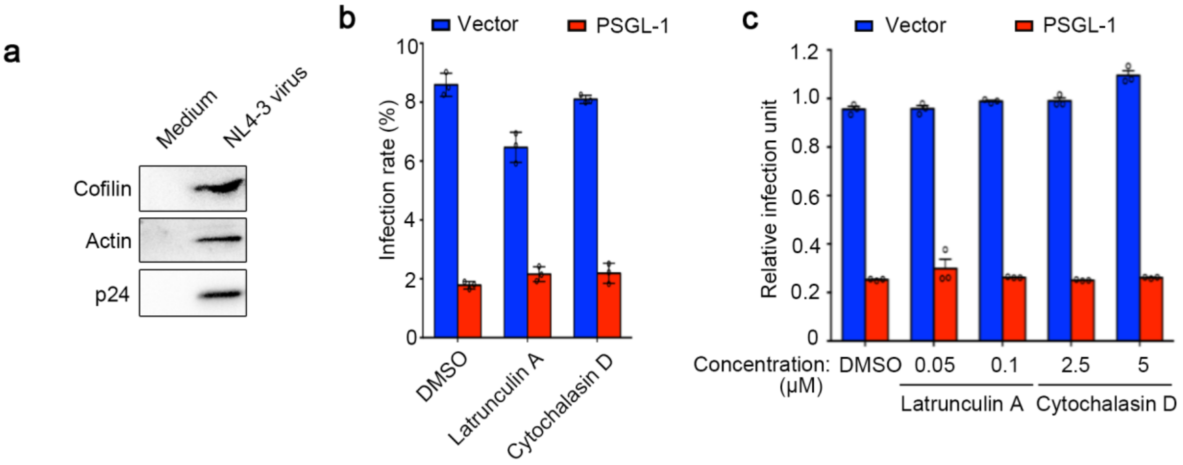

Supplementary information, Fig. S3 PSGL-1 stabilizes F-actin in HIV virions. a Western blotting analysis shows nascent HIV-1 virions contain abundant cofilin and actin. **b** Vpr-BlaM containing NL4-3 viruses generated from 283T cells with or without PSGL-1 overexpression were normalized with p24 ELISA. The viruses were used to infect cells treated with latrunculin A or cytochalasin D or DMSO control. The concentrations of the compounds are equal to the higher concentration of each drug in **Fig.3d**. Two hours after infection, the cells were incubated with β-lactamase substrate overnight before being fixed and analyzed by FACs. **c** TZM-bl cells were treated with actin inhibitors latrunculin A or cytochalasin D or DMSO control at the concentration that is equal to the final concentration of each drug in the cell medium as in **Fig.3e**. NL4-3 viruses generated from 283T cells with or without PSGL-1 overexpression were normalized with p24 ELISA and used to infect the TZM-bl cells pretreated with the indicated compounds. Two days after infection, the infection rate was quantitated using luciferase assay.

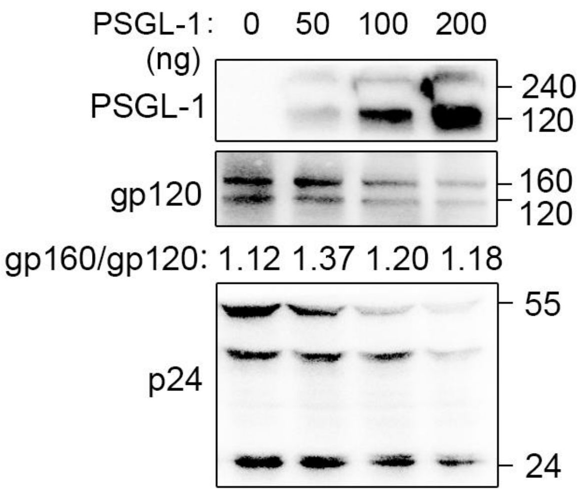

Supplementary information, Fig. S4 PSGL-1 does not affect Env or Gag processing. 293T cells were transfection with 1 μg pNL4-3 and different amounts of pCMV-PSGL-1 for 48 h before being lysed for Western blotting analysis. Empty vector was used to normalize the total DNA transfect.

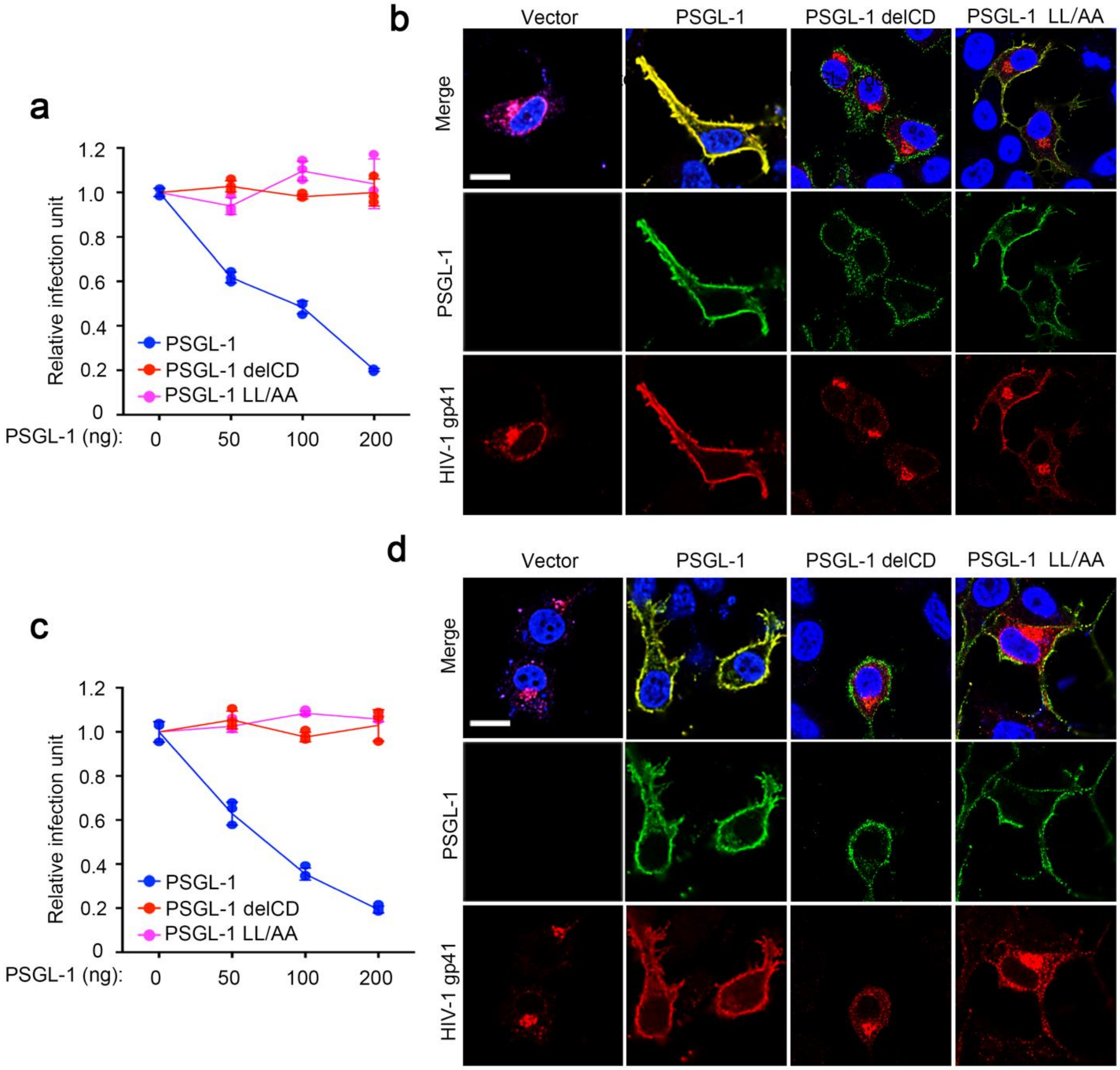

Supplementary information, Fig. S5 PSGL-1 inhibits R5-tropic virions infectivity and interacts with R5-tropic gp41. TZM-bl cells were infected with virions harvested from 293T cells transfected with two different R5-tropic HIV plasmids pYU2 (**a**) or pNL(AD8) (**c**) and different amounts of plasmids expressing PSGL-1. Empty vector was used to normalize the total transfected DNA. The virions were normalized by p24 ELISA. The infection rates were quantitated with luciferase assays. N=3. 293T cells transfected with pYU2 (**b**) or pNL(AD8) (**d**) and PSGL-1 or an empty vector were fixed and stained with anti-gp41 (red), anti-PSGL-1 (green) antibodies and DAPI (blue). Scale bar: 5μm.

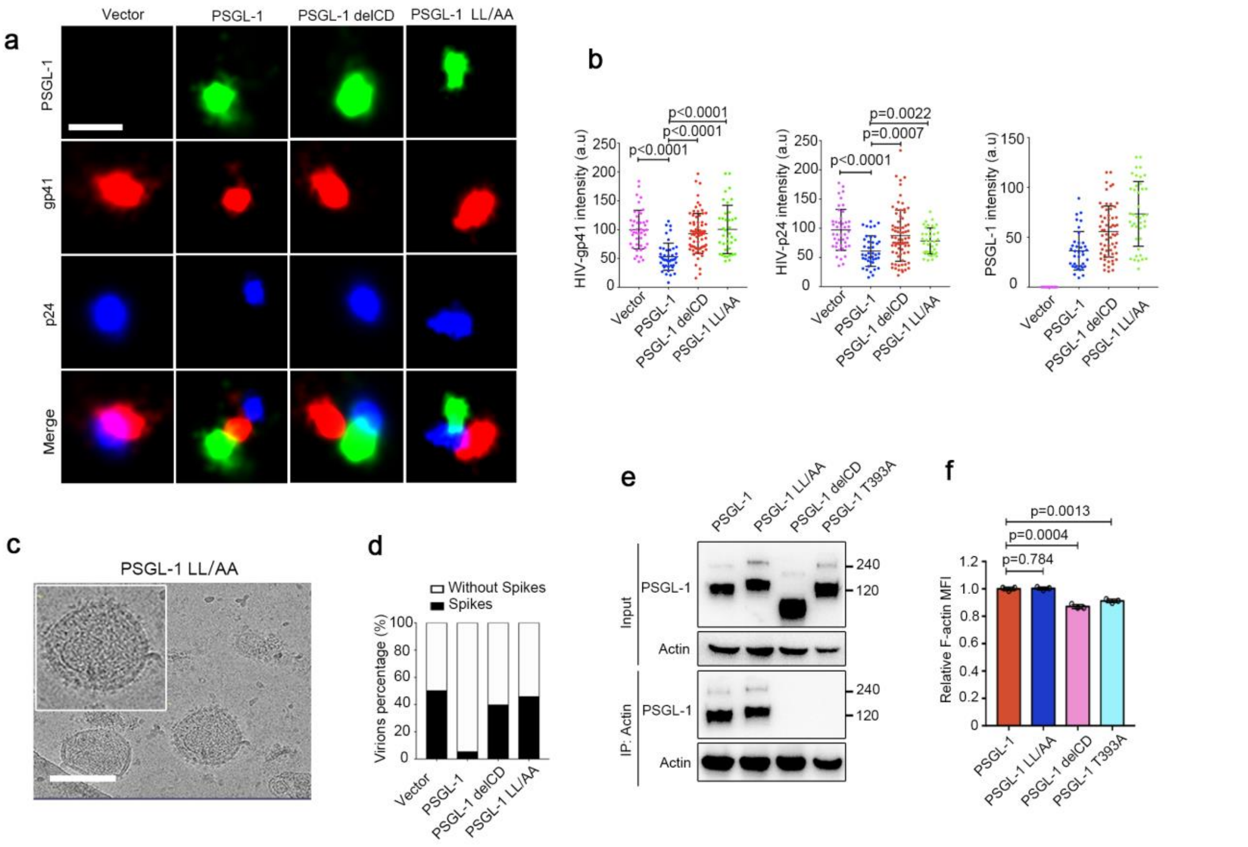

Supplementary information, Fig. S6 PSGL-1 LL/AA is deficient in gp41 interaction, but not in actin-binding. a, **b** Virions from producer 293T cells transfected with PSGL-1, PSGL-1 delCD or PSGL-1 LL/AA or an empty vector were pelleted through 20% sucrose cushion were fixed and stained for STORM imaging. Representative images are shown in **a** and the quantifications of images are shown in **b**. The quantifications were analyzed together with the data in **Fig. 4g** for comparison. **c**, **d** Concentrated virions harvested from 293T cells transfected with pNL4-3 and PSGL-1 LL/AA were analyzed by cryo-EM analysis. Scale bar: 100nm. Representative images were shown in **c** and quantification of images of virions shown in **d**. Scale bar: 100 nm. The quantifications were analyzed together with the data in **Fig. 4i** for comparison. **e** 293T cells were transfected with plasmids expressing PSGL-1, PSGL-1 delCD, PSGL-1 LL/AA and PSGL-1 T393A. Two days after the transfection, PSGL-1 were immunoprecipitated with protein A agarose beads with anti-Actin antibody. The cell lysates and the precipitated proteins were analyzed by Western blotting. **f** Jurkat cells transfected with different PSGL-1 constructs for 48 h then for phalloidin staining and F-actin quantification by FACS. $n = 3$.

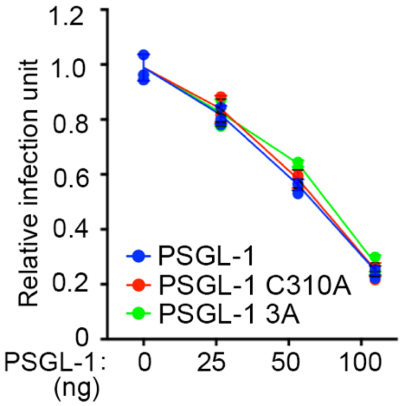

Supplementary information, Fig. S7 PSGL-1's dimerization-deficient mutation and gap co-clustering-deficient mutations have no effect on its anti-viral activity. a TZM-bl cells were infected with virions harvested from 293T cells transfected with pNL4-3 plasmids and different amounts of plasmids expressing PSGL-1 or PSGL-1 C336A mutant (dimerization-deficient), PSGL-1 3A mutant (gap co-clustering-deficient**)**. The virions were normalized by p24 ELISA before the infection. The infection rates were quantitated with luciferase assays. $n = 3$.

Supplementary Movie legends:

Supplementary Movie S1. F-actin+cofilin+GST.avi and **Movie S2. F-actin+cofilin+CD- GST.avi:**

Purified PSGL-1 cytoplasmic domain in fusion with GST (CD-GST) or GST was mixed with purified and in vitro polymerized F-actin labeled 5-(and-6)-carboxytetramethylrhodamine-succinimidylester for 30 min then the mixtures injected into the flow cell coated with 25 nM N-ethylmaleimidemyosin. Cofilin was injected into the chamber at 10 μM final concentration. Single actin filaments were observed by TIRF illumination with an Olympus IX81 microscope equipped with a 100x oil objective (1.49 NA). Images were collected for 5min with an interval of 3s. The movies were generated with a compression rate of 7 frames/second.
